# Associations between ABO blood groups and pancreatic ductal adenocarcinoma: influence on resection status and survival

**DOI:** 10.1002/cam4.1097

**Published:** 2017-05-29

**Authors:** Khadija El Jellas, Dag Hoem, Kristin G Hagen, May Britt Kalvenes, Sura Aziz, Solrun J Steine, Heike Immervoll, Stefan Johansson, Anders Molven

**Affiliations:** ^1^ Gade Laboratory for Pathology Department of Clinical Medicine University of Bergen Bergen Norway; ^2^ Department of Pathology Haukeland University Hospital Bergen Norway; ^3^ Center for Medical Genetics and Molecular Medicine Haukeland University Hospital Bergen Norway; ^4^ Department of Gastrointestinal Surgery Haukeland University Hospital Bergen Norway; ^5^ Department of Immunology and Transfusion Medicine Haukeland University Hospital Bergen Norway; ^6^ KG Jebsen Center for Diabetes Research Department of Clinical Science University of Bergen Bergen Norway

**Keywords:** ABO, blood group, FUT2, glycosyltransferase, pancreatic ductal adenocarcinoma, risk factor

## Abstract

Both serology‐based and genetic studies have reported an association between pancreatic cancer risk and ABO blood groups. We have investigated this relationship in a cohort of pancreatic cancer patients from Western Norway (*n *=* *237) and two control materials (healthy blood donors, *n* = 379; unselected hospitalized patients, *n *=* *6149). When comparing patient and blood donor *ABO* allele frequencies, we found only the A_1_ allele to be associated with significantly higher risk for pancreatic ductal adenocarcinoma (PDAC) (23.8% vs. 17.9%; OR = 1.43, *P *=* *0.018). Analyzing phenotypes, blood group A was more frequent among PDAC cases than blood donors (50.8% vs. 40.6%; OR = 1.51, *P *=* *0.021), an enrichment fully explained by the A_1_ subgroup. Blood group O frequency was lower in cases than in blood donors (33.8% vs. 42.7%; OR = 0.69, *P *=* *0.039). This lower frequency was confirmed when cases were compared to hospitalized patients (33.8% vs. 42.9%; OR = 0.68, *P *=* *0.012). Results for blood group B varied according to which control cohort was used for comparison. When patients were classified according to surgical treatment, the enrichment of blood group A was most prominent among unresected cases (54.0%), who also had the lowest prevalence of O (28.7%). There was a statistically significant better survival (*P *=* *0.04) for blood group O cases than non‐O cases among unresected but not among resected patients. Secretor status did not show an association with PDAC or survival. Our study demonstrates that pancreatic cancer risk is influenced by ABO status, in particular blood groups O and A_1_, and that this association may reflect also in tumor resectability and survival.

## Introduction

Pancreatic cancer is one of the most dreaded malignant diseases. It ranks the fourth most common cause of cancer‐related death in many Western countries and has a remarkably dismal prognosis with a 5‐year survival rate of <5% 1. The manifestation of symptoms occurs relatively late in the disease process. Most pancreatic cancer cases are therefore diagnosed at an unresectable stage and only 15–20% of the patients undergo surgical resection, which is the only potentially curative treatment.

Histologically, the large majority of pancreatic cancer cases are exocrine and classified as pancreatic ductal adenocarcinoma (PDAC), in which the characteristic morphological pattern consists of abundant duct‐like neoplastic structures embedded in a dense desmoplastic stroma 2. Accepted risk factors for PDAC include advanced age, cigarette smoking 3, and long‐standing chronic pancreatitis 4, whereas some studies also have implicated *Helicobacter pylori* infection 5 and diabetes mellitus 6. In addition, inherited susceptibility plays a role in the disease, both as high‐risk gene variants in the context of familial cancer syndromes 7 and as the presence of variants with modest effect, usually discovered by genome‐wide association studies (GWAS) 8.

In a landmark GWAS paper, the Pancreatic Cancer Cohort Consortium (PanScan) reported that the statistically most significant variants associating with pancreatic cancer risk belonged to the *ABO* locus on chromosome 9q34 9. *ABO* codes for a glycosyltransferase that gives rise to the histo‐blood group antigens of the ABO system. Single‐nucleotide polymorphisms (SNPs) of this gene determine the specificity of the enzyme 10, 11. Hence, by adding either *N*‐acetyl‐d‐galactosamine or d‐galactose to the precursor H antigen, the A and B glycosyltransferases produce A or B antigens, respectively, on cellular surfaces and secretions. A frequent *ABO* variant is a one‐base pair deletion that inactivates the encoded enzyme, leaving the H antigen unaltered and corresponding to the O phenotype 10. This deletion is in strong linkage disequilibrium with the T allele of SNP rs505922, which was identified as being associated with decreased susceptibility for pancreatic cancer 9. Accordingly, individuals with blood group O have a lower risk for this disease than those with other blood groups 12.

There are two major subtypes of the *ABO* allele determining the blood group A, namely *A*_*1*_ and *A*_*2*_. Data from PanScan demonstrated that, among all common *ABO* variants, the greatest risk of pancreatic cancer was conferred by the *A*_*1*_ allele 13 which gives rise to the ABO protein with highest enzymatic activity 14. This finding suggests that it is the glycosyltransferase activity itself that is linked to cancer risk rather than actions of other nearby genes on chromosome 9q34. Somewhat surprisingly though, the association between blood group and pancreatic cancer was not influenced by the secretor phenotype, that is, a person's ability to secrete A, B, and H antigens into body fluids 13. This property is determined by *FUT2*, the gene encoding the glycosylating enzyme fucosyltransferase 2.

The initial reports 9, 12, 13 were followed up by genetic studies in various populations that confirmed the influence of ABO blood group alleles on pancreatic cancer risk 15, 16, 17. The finding that blood group O confers protection was also consistent with older papers that had reported an association between ABO phenotype and gastrointestinal cancers including pancreatic cancer 18, 19, 20. A meta‐analysis including over 20 studies, both genetic and serology‐based, concluded that all non‐O blood groups have elevated risks for pancreatic cancer as compared with the O phenotype 16.

In this study, we aimed at evaluating the link between pancreatic cancer and ABO histo‐blood groups in patients from Norway, a population in which this association has not yet been investigated. Our patients were carefully characterized to exclude non‐PDAC cases and also classified according to tumor resectability and survival. Two different sets of controls were included for statistical comparisons.

## Materials and Methods

### Study population

The study was performed according to the Helsinki Declaration and all patients gave their written informed consent. The project was approved by the Regional Ethical Committee of Western Norway. The patient cohort consisted of 237 cases of pancreatic adenocarcinoma seen at Haukeland University Hospital, Bergen, Norway between the years 1998 and 2012 (Table 1). Medical records, pathology reports, and/or tissue sections were examined by two pathologists (HI and SA) for diagnosis confirmation. Final classification resulted in 195 PDAC cases and 42 other adenocarcinomas located within the pancreas. The latter group consisted of intraductal papillary mucinous neoplasm (5 cases) or mucinous cystic neoplasm with malignant component (1 case), intrapancreatic adenocarcinoma of the ampulla/papilla of Vateri (19 cases) or of ductus choledochus (9 cases), and unspecified adenocarcinoma of the pancreas (8 cases).

**Table 1:** Overview of pancreatic cancer patients and controls included in the study

Cohort	Total *n*	Females			Males		
		*n*	%	Median age	*n*	%	Median age
Cases							
All pancreatic adenocarcinoma cases	237	119	50.2	69	118	49.8	69
Pancreatic ductal adenocarcinoma (PDAC)	195	97	49.7	69	98	50.3	69
Resected	108	50	46.3	70	58	53.7	68
Not resected	87	47	54.0	70	40	46.0	67
Other adenocarcinomas^a^	42	22	52.4	67	20	47.6	68
Controls							
DNA‐typed blood donors	379	189	49.9	39	190	50.1	44
Serotyped hospital patients	6149	2805	45.6	66	3344	54.4	64

a See Materials and Methods for description.

For the statistical comparison, two different control groups were employed (Table 1). One consisted of 379 healthy blood donors from Haukeland University Hospital (49.9% females) that were genotyped in the same way as the cases. The other control group contained 6149 patients (45.6% females) born before 1.1.1970 and admitted to the same hospital during a randomly chosen period of six consecutive months in 2007. All patients had been blood‐typed by serological means as part of their health care. No selection with regard to diagnosis was done.

### DNA extraction and genotyping

EDTA‐blood, frozen tissue and formalin‐fixed paraffin‐embedded (FFPE) tissue blocks were used for DNA extraction from the 237 pancreatic adenocarcinoma cases. DNA from frozen buffy coats from EDTA‐blood (195 cases) were purified using MagAttract DNA Blood Midi M48 kit on the BioRobot M48 workstation (both from Qiagen, Hilden, Germany) or manually processed with the E.Z.N.A DNA extraction kit (Omega Bio‐Tek, Norcross, GA, USA), according to the manufacturers’ protocol. For 38 cases, only FFPE tissue samples were available; 10‐micron sections were then sliced and subjected to manual deparaffinization with xylene, followed by ethanol washes before overnight incubation at 56°C with Proteinase K (Qiagen) and processing with the E.Z.N.A. DNA extraction kit. For the final four cases, only fresh‐frozen tissue samples were available. These were incubated directly with Proteinase K and then processed as the FFPE samples. DNA from the blood donor controls was isolated from EDTA‐blood buffy coats using the same purification system as for the patient blood samples.

Genotyping was performed using TaqMan predesigned genotyping assays (Cat. No. 4351379; Applied Biosystems, Foster City, CA). Each sample was tested for three common SNPs at the *ABO* locus (Table S1): rs8176704 (intron 3) for the A_2_ allele (Assay ID: C_30336657_10), rs8176746 (exon 7) for the B allele (Assay ID: C_25610772_20), and rs505922 (intron 1) for the O allele (Assay ID: C_2253769_10). The samples were also screened for the *FUT2* variant rs601338 (Assay ID: C_2405292_10), which determines secretor status of ABH antigens.

The genotyping assays were performed on the 7900 Fast Real‐time PCR System with the corresponding 7900 Fast System SDS 2.4 Software (Applied Biosystems). Positive and negative controls were included to ensure appropriate clustering. Each assay was performed using 10 ng template DNA, TaqMan Universal Master Mix buffer (Applied Biosystems), and 20x primer and probe mix as recommended by the manufacturer. Thermal cycling was performed by first activating the DNA polymerase at 95°C for 10 min and then running 40 amplification cycles, each consisting of denaturation at 92°C for 15 sec and combined annealing/extension at 60°C for 1 min.

### Quality control

Misclassification of ABO genotypes was expected to be minimal, as genotyping results from the three different ABO SNPs matched the haplotype phasing of the *ABO* gene (Table 1 in 13) for all analyzed samples. In addition, we compared our genotyping results for 40 cases (20 extracted from blood and 20 from FFPE tissues) with serologically determined ABO status as stated in the patients’ medical records. The concordance rate was 100%. Genotyping quality for the blood donor control group was assessed by testing for Hardy–Weinberg equilibrium using the Haploview Software 21. The distribution of genotypes was as expected from the SNP frequencies.

### Statistical analysis

Each SNP was tested under various genetic models (dominant, codominant, recessive, additive) using the software PLINK ( http://pngu.mgh.harvard.edu/~purcell/plink). Assessment of differences in genotype or phenotype distributions between cases and controls was carried out by the two‐tailed Fisher`s exact test or Pearson`s chi‐square test. Odds ratios (OR) and 95% confidence intervals (CI) were calculated using two‐by‐two contingency table analysis on the SISA webpage ( http://www.quantitativeskills.com/sisa). Survival analysis was performed using the software package STATISTICA version 12 (StatSoft, Tulsa, OK). The Product–Limit (Kaplan–Meier) Analysis Module was employed for comparing survival between groups by log‐rank test of significance. Survival times versus cumulative proportion surviving, according to breakdown by blood group, were plotted. In all tests, *P* ≤ 0.05 was chosen for statistical significance.

## Results

### Patient characteristics

From our biobank of patients with pancreatic tumors, we initially selected the patients diagnosed with adenocarcinoma of the exocrine gland. This cohort consisted of 237 cases (50.2% females), with a median age at diagnosis of 69 years in both sexes (Table 1). We reviewed all cases to identify those that were consistent with a diagnosis of PDAC (*n *=* *195, 82.3%). The cases were also classified according to whether or not the tumor had been judged resectable at the time of diagnosis (Table 1).

### Association between ABO blood group and pancreatic cancer risk

The genotype frequencies for all adenocarcinoma cases and blood donor controls are given in Table S2. In both groups, the most common ABO genotypes were A_1_O and OO, and the least frequent were BB and A_2_A_2_. We first compared allele frequencies of A_1_, A_2_, B, and O in the whole adenocarcinoma cohort with frequencies observed in the blood donors (Table 2, ‘All cases’). The A_1_ allele frequency was higher among the patients (22.4% vs. 17.9%) but the difference did not quite reach statistical significance (*P *=* *0.057). When the analysis was limited to PDAC cases only, there was a significant difference in A_1_ frequency (23.8% vs. 17.9%; OR *= *1.43, CI = 1.06–1.93; *P = *0.018). Interestingly, the A_2_ frequency appeared almost identical between the groups compared (7.6–7.8%). The B and O allele frequencies varied, but were not statistically different.

**Table 2:** *ABO* and *FUT2* allele frequencies of blood donor controls compared with pancreatic cancer cases

Allele	Controls (*n *=* *758)	All cases (*n *=* *474)			PDAC cases only (*n *=* *390)		
	%	%	*P*	OR (95% CI)	%	*P*	OR (95% CI)
*ABO*							
A_1_	17.9	22.4	0.057	1.32 (0.99–1.75)	23.8	**0.018**	1.43 (1.06–1.93)
A_2_	7.8	7.6	1.000	0.97 (0.63–1.50)	7.7	1.000	0.99 (0.63–1.56)
B	8.7	7.6	0.491	0.86 (0.56–1.32)	7.7	0.556	0.87 (0.56–1.37)
O	65.6	62.4	0.266	0.87 (0.69–1.11)	60.8	0.109	0.81 (0.63–1.05)
*FUT2*							
Se	51.5	51.5	1.000	1.00 (0.79–1.26)	52.1	0.847	1.02 (0.80–1.31)
Se^0^	48.5	48.5	1.000	1.00 (0.79–1.26)	47.9	0.847	0.98 (0.77–1.25)

n, number of genotyped alleles; PDAC, pancreatic ductal adenocarcinoma; *P*,* P* ‐value from chi‐square test (df = 1); OR (95% CI), odds ratio (95% confidence interval). Significant *P*‐value is shown in bold face.

For the further analyses, we restricted our analysis to the PDAC cases only. We deduced ABO phenotypes from the genotype data of the cases and the blood donors. The blood group distributions are shown in Table 3. The blood group A prevalence was clearly different (50.8% vs. 40.6%; OR = 1.51, CI = 1.06–2.13; *P = *0.021,) and, in keeping with the data of Table 2, the subgroup A_1_ frequencies fully explained the observed difference (42.6% vs. 29.3%; OR = 1.79, CI = 1.25–2.56; *P = *0.001). Moreover, the prevalence of blood group O was lower in cases than in controls (33.8% vs. 42.7%; OR = 0.69, CI = 0.48–0.98; *P = *0.039). Blood group B did not show a statistically significant difference in distribution. Neither did blood group AB, although in this case, the number of subjects was too small for meaningful comparisons to be made.

**Table 3:** ABO blood group and secretor phenotype frequencies of blood donor controls compared with PDAC cases

Phenotype	Controls (*n *=* *379)%	PDAC cases (*n *=* *195)			PDAC cases according to resection status					
					Resected (*n *=* *108)			Unresected (*n *=* *87)		
		%	*P*	OR (95% CI)	%	*P*	OR (95% CI)	%	*P*	OR (95% CI)
*ABO*										
A	40.6	50.8	**0.021**	1.51 (1.06–2.13)	48.1	0.163	1.36 (0.88–2.09)	54.0	**0.023**	1.72 (1.07–2.74)
A_1_	29.3	42.6	**0.001**	1.79 (1.25–2.56)	40.7	**0.024**	1.66 (1.07–2.59)	44.8	**0.005**	1.96 (1.22–3.16)
A_2_	11.3	8.2	0.241	0.70 (0.38–1.27)	7.4	0.238	0.63 (0.29–1.37)	9.2	0.562	0.79 (0.36–1.75)
B	12.1	12.8	0.814	1.06 (0.63–1.79)	11.1	0.771	0.91 (0.46–1.78)	14.9	0.478	1.27 (0.65–2.47)
AB^a^	4.5	2.6	0.359	0.56 (0.20–1.54)	2.8	0.586	0.61 (0.18–2.12)	2.3	0.548	0.50 (0.11–2.21)
O	42.7	33.8	**0.039**	0.69 (0.48–0.98)	38.0	0.374	0.82 (0.53–1.27)	28.7	**0.016**	0.54 (0.33–0.90)
*FUT2*										
Secretor	77.6	76.4	0.753	0.94 (0.62–1.41)	72.2	0.248	0.74 (0.46–1.22)	81.6	0.410	1.28 (0.71–2.32)
Non‐secretor	22.4	23.6	0.753	1.07 (0.71–1.61)	27.8	0.248	1.33 (0.82–2.16)	18.4	0.410	0.78 (0.43–1.41)

PDAC, pancreatic ductal adenocarcinoma; *P*,* P* ‐value from chi‐square test (df=1); OR (95% CI), odds ratio (95% confidence interval). Significant *P* ‐values are shown in bold face.

a *P* ‐values from two‐tailed Fisher`s exact test.

Healthy blood donors may not always serve as an optimal control group in case–control studies 22. We therefore collected information on ABO blood group distribution from a large control cohort of unselected hospitalized patients (see Materials) from the same geographical region as our pancreatic cancer patients. The subgroup A_1_/A_2_ distribution was not known for the hospitalized patient cohort. Notably, the hospitalized patients had a significantly higher blood group A prevalence than the blood donors (45.8% vs. 40.6%; OR = 1.24, CI = 1.00–1.53; *P = *0.049). Similarly, blood group B was significantly less frequent (7.7% vs. 12.1%; OR *= *0.60, CI = 0.44–0.83; *P = *0.002). Blood group O had very similar prevalence in the two control groups (42.7% and 42.9%).

When the PDAC blood group distribution was compared with that of the hospitalized patients, the enrichment of blood group A among the PDAC cases no longer reached statistical significance (50.8% vs. 45.8%; OR = 1.22, CI = 0.92–1.62; *P = *0.173) (Table 4). On the other hand, the blood group B difference was now significant (12.8% vs. 7.7%; OR = 1.76, CI = 1.15–2.71; *P = *0.009). The lower frequency of blood group O in the PDAC cohort remained significant with almost identical odds ratio (33.8% vs. 42.9%; OR = 0.68, CI = 0.50–0.92; *P *=* *0.012).

**Table 4:** ABO phenotypic frequencies of hospital patient controls compared with PDAC cases

Blood types	Controls (*n *=* *6149) %	PDAC cases (*n *=* *195)			PDAC cases according to resection status					
					Resected (*n *=* *108)			Unresected (*n *=* *87)		
		%	*P*	OR (95% CI)	%	*P*	OR (95% CI)	%	*P*	OR (95% CI)
A	45.8	50.8	0.173	1.22 (0.92–1.62)	48.1	0.632	1.10 (0.75–1.61)	54.0	0.128	1.39 (0.91–2.12)
B	7.7	12.8	**0.009**	1.76 (1.15–2.71)	11.1	0.188	1.50 (0.82–2.75)	14.9	**0.012**	2.11 (1.16–3.83)
AB^a^	3.5	2.6	0.690	0.72 (0.29–1.76)	2.8	1.000	0.78 (0.25–2.47)	2.3	0.771	0.64 (0.16–2.62)
O	42.9	33.8	**0.012**	0.68 (0.50–0.92)	38.0	0.301	0.81 (0.55–1.20)	28.7	**0.008**	0.54 (0.34–0.86)

PDAC, pancreatic ductal adenocarcinoma; *P*,* P*‐value from chi‐square test (df=1); OR (95% CI), odds ratio (95% confidence interval). Significant *P* ‐values are shown in bold face.

a *P*‐values from two‐tailed Fisher`s exact test

### Tumor resectability

To further explore the association between blood group frequencies and PDAC, the patients were stratified into two subgroups: those who had their pancreatic tumor resected and those who were considered surgically unresectable at the time of cancer diagnosis (Table 1). The latter group consisted of patients with locally advanced tumors with encasement of adjacent large blood vessels (Clinical stage III) or with metastatic disease at the time of diagnosis (Clinical stage IV) 23. We observed that the blood group A and subgroup A_1_ prevalences were highest among the unresected cases (54.0% and 44.8%, respectively) and both were significantly different from the frequencies found in blood donors (A: OR = 1.72, CI = 1.07–2.74, *P = *0.023; A_1_: OR = 1.96, CI = 1.22–3.16, *P = *0.005) (Table 3). Moreover, the unresected cases had the lowest frequency of blood group O (28.7% vs. 42.7%; OR = 0.54, CI = 0.33–0.90; *P = *0.016). A comparison with the cohort of hospitalized patients (Table 4) revealed significant differences for the unresected patients, both with regard to blood group B (14.9% vs. 7.7%; OR = 2.11, CI = 1.16–3.83; *P = *0.012) and blood group O (28.7% vs. 42.9%; OR = 0.54, CI = 0.34–0.86; *P = *0.008).

### Survival

**Figure 1:**
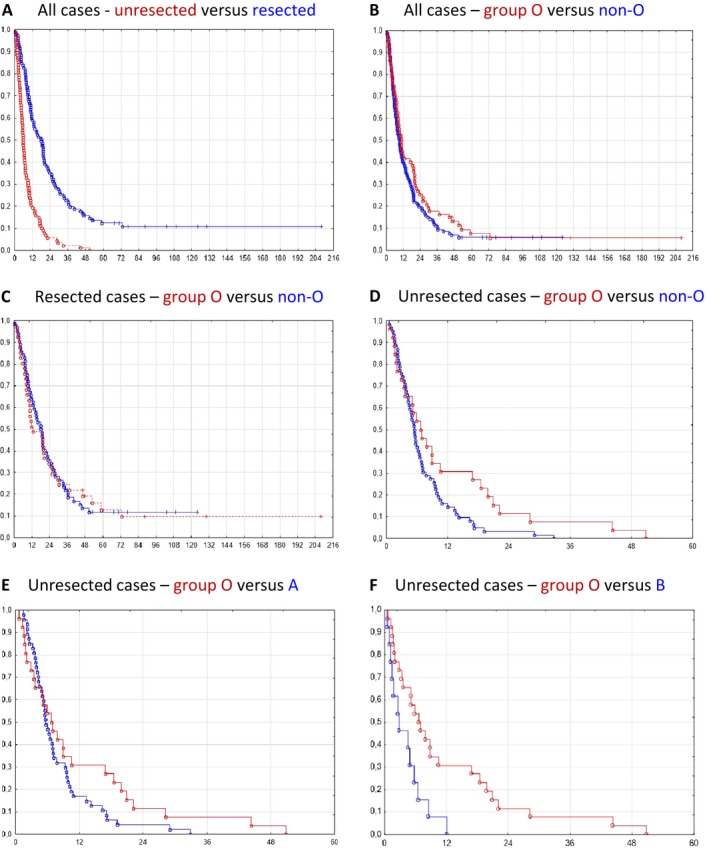
Cumulative proportion survival (Kaplan–Meier) plot for the 195 pancreatic ductal adenocarcinomas according to breakdown by resection status and blood group phenotype. (A)‐(F) Pairwise comparison of subgroups as specified in the heading of each panel. The observed survival times (months along the *X*‐axis) are indicated by circles (complete) or crosses (censored observations). In panel A, survival curves for resected and unresected cases are shown by a blue and a red line, respectively. In all other panels, survival curves for blood group O and the comparison group are shown by a red and blue line, respectively.

Data on survival after time of diagnosis was available for all PDAC cases. As expected, survival was significantly better among resected than among unresected cases (median survival 18.1 months vs. 5.6 months, respectively; *P < *0.001) (Fig. 1A). Given that blood group is a risk factor for PDAC and that it also may influence resection status, we also analyzed survival according to ABO phenotype. When all 195 patients were classified as O or non‐O cases, survival of the two groups was not significantly different (median 10.4 vs. 9.3 months, respectively; *P = *0.23) (Fig. 1B). We then looked at resected and unresected cases separately. Among the resected cases, survival did not differ between patients with O and non‐O blood group (*P = *0.93) (Fig. 1C). However, in the group of unresected cases, patients with blood group O survived longer than non‐O patients (median 6.7 vs. 5.5 months, respectively; *P = *0.04) (Fig. 1D). When the non‐O cases were split into blood group A or B and compared to the O cases, the difference in survival reached significance for blood group B (median 2.7 months; *P = *0.03), but not for A (median 5.6 months; *P = *0.14) (Fig. 1E–F). Although it should be noted that the number of A_2_ cases is relatively small (16/195 cases), we also examined whether there was a survival difference between patients of blood group A_1_ and A_2_. Neither among all cases nor when cases were classified according to resection status was any statistically significant difference observed (data not shown).

### Secretor status

Because the secretion of soluble H antigen is associated with susceptibility to multiple pathogens through adherence to the gastrointestinal mucosa we looked at the secretor phenotype determined by the rs601338 *FUT2* polymorphism (Tables S1, S2). Consistent with another study 13, there were no significant differences in prevalence of alleles or phenotypes when compared with the blood donor controls (Tables 2, 3). We further checked whether *FUT2* could have an effect on resection status by comparing the groups of resected and unresected PDAC cases in Table 3 directly against each other. The difference in secretor status was not significant (72.2% vs. 81.6%; OR=0.59, CI=0.30–1.16; *P *=* *0.124). Finally, we examined if secretor status might associate with survival in our patient cohort. No such association was seen (Fig. S1).

## Conflict of Interest

None declared.

## Supporting information


**Figure S1**. Cumulative proportion survival (Kaplan–Meier) plot for the 195 pancreatic ductal adenocarcinomas according to breakdown by *FUT2* secretor phenotype and resection status.

**Table S1.** SNPs used for *ABO* and *FUT2* allele genotyping.
**Table S2.** *ABO* and *FUT2* genotype frequencies of blood donor controls and pancreatic cancer cases.
