# Supplementary material for: Associations between ABO blood groups and pancreatic ductal adenocarcinoma: influence on resection status and survival
Source: Cancer Med. 2017 May 29;6(7):1531–40. doi: 10.1002/cam4.1097 (PMC5504338; doi:10.1002/cam4.1097)

Supplementary Figure 1

A: All cases - Secretor vs. Non-secretor

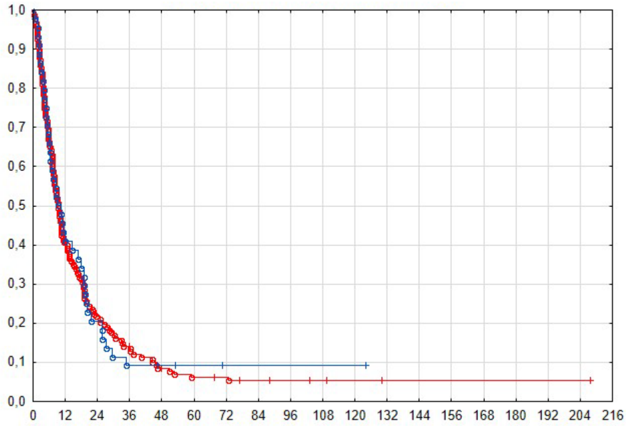

B: Resected cases - Secretor vs. Non-secretor

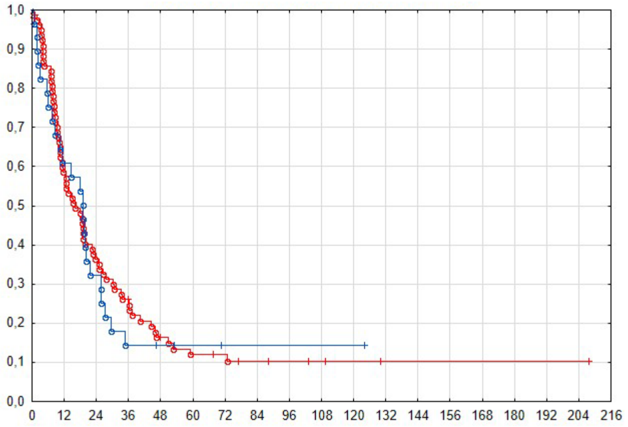

C: Unresected cases - Secretor vs. Non-secretor

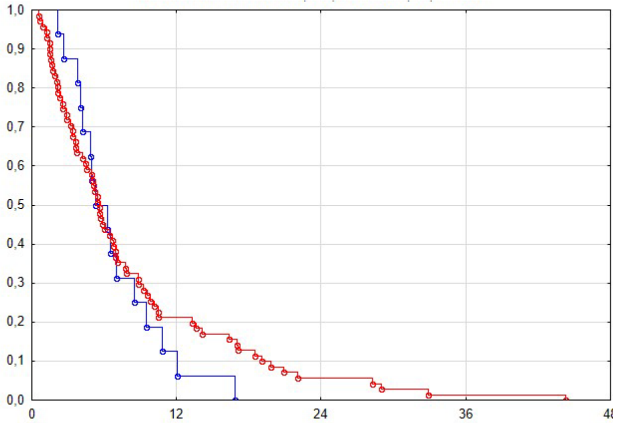

Supplement: Supplementary file 1 — Figure S1. Cumulative proportion survival (Kaplan–Meier) plot for the 195 pancreatic ductal adenocarcinomas according to breakdown by FUT2 secretor phenotype and resection status. [file CAM4-6-1531-s001.pdf]
