# Supplementary material for: Associations between ABO blood groups and pancreatic ductal adenocarcinoma: influence on resection status and survival
Source: Cancer Med. 2017 May 29;6(7):1531–40. doi: 10.1002/cam4.1097 (PMC5504338; doi:10.1002/cam4.1097)
Supplement: Supplementary file 2 — Table S1. SNPs used for ABO and FUT2 allele genotyping. Table S2. ABO and FUT2 genotype frequencies of blood donor controls and pancreatic cancer cases. [file CAM4-6-1531-s002.pdf]

Supplementary Table 1. SNPs used for *ABO* and *FUT2* allele genotyping

	ABO SNPs			***FUT2 SNP***
Allele	rs8176704	rs8176746	rs505922	rs601338
A_1	G	C	C	-
A_2	A	C	C	-
B	G	A	C	-
O	G	C	T	-
Se	-	-	-	G
Se^0	-	-	-	A

Se and Se^0 refer to secretor and non-secretor allele, respectively

Supplementary Table 2. *ABO* and *FUT2* genotype frequencies of blood donor controls and pancreatic cancer cases

	Controls (n =379)		All cases (n =237)		PDAC cases only (n =195)	
**Genotype**	***n***	**%**	***n***	**%**	***n***	**%**
ABO						
A_1A_1	15	4.0	6	2.5	6	3.1
A_1A_2	5	1.3	14	5.9	13	6.7
A_2A_2	4	1.1	0	0.0	0	0.0
A_1O	91	24.0	75	31.6	64	32.8
A_2O	39	10.3	20	8.4	16	8.2
BB	3	0.8	0	0.0	0	0.0
BO	43	11.3	29	12.2	25	12.8
A_1B	10	2.6	5	2.1	4	2.1
A_2B	7	1.8	2	0.8	1	0.5
OO	162	42.7	86	36.3	66	33.8
FUT2						
SeSe	96	25.3	64	27.0	64	27.0
$SeSe^0$	198	52.2	116	48.9	116	48.9
Se^0Se^0	85	22.4	57	24.1	57	24.1
